# Phenolic extract from oleaster (*Olea europaea* var. *Sylvestris*) leaves reduces colon cancer growth and induces caspase-dependent apoptosis in colon cancer cells via the mitochondrial apoptotic pathway

**DOI:** 10.1371/journal.pone.0170823

**Published:** 2017-02-17

**Authors:** Wafa Zeriouh, Abdelhafid Nani, Meriem Belarbi, Adélie Dumont, Charlotte de Rosny, Ikram Aboura, Fatima Zahra Ghanemi, Babar Murtaza, Danish Patoli, Charles Thomas, Lionel Apetoh, Cédric Rébé, Dominique Delmas, Naim Akhtar Khan, François Ghiringhelli, Mickael Rialland, Aziz Hichami

**Affiliations:** 1 Laboratory of Natural Products, Aboubekr Belkaid University, Tlemcen, Algeria; 2 Department of Natural and Life Sciences, African University Ahmed Draia, Adrar, Algeria; 3 INSERM U1231, Université de Bourgogne Franche-Comté, Dijon, France; 4 Centre Georges François Leclerc, Dijon, France; Universitat des Saarlandes, GERMANY

## Abstract

Dietary polyphenols, derived from natural products, have received a great interest for their chemopreventive properties against cancer. In this study, we investigated the effects of phenolic extract of the oleaster leaves (PEOL) on tumor growth in mouse model and on cell death in colon cancer cell lines. We assessed the effect of oleaster leaf infusion on HCT116 (human colon cancer cell line) xenograft growth in athymic nude mice. We observed that oleaster leaf polyphenol-rich infusion limited HCT116 tumor growth in vivo. Investigations of PEOL on two human CRC cell lines showed that PEOL induced apoptosis in HCT116 and HCT8 cells. We demonstrated an activation of caspase-3, -7 and -9 by PEOL and that pre-treatment with the pan-caspase inhibitor, N-benzyloxycarbonyl-Val-Ala-Asp-fluoromethylketone (z-VAD-fmk), prevented PEOL-induced cell death. We observed an involvement of the mitochondrial pathway in PEOL-induced apoptosis evidenced by reactive oxygen species (ROS) production, a decrease of mitochondrial membrane potential, and cytochrome c release. Increase in intracellular Ca^2+^ concentration induced by PEOL represents the early event involved in mitochondrial dysfunction, ROS-induced endoplasmic reticulum (ER) stress and apoptosis induced by PEOL, as ruthenium red, an inhibitor of mitochondrial calcium uptake inhibited apoptotic effect of PEOL, BAPTA/AM inhibited PEOL-induced ROS generation and finally, N-acetyl-L-cysteine reversed ER stress and apoptotic effect of PEOL. These results demonstrate that polyphenols from oleaster leaves might have a strong potential as chemopreventive agent in colorectal cancer.

## Introduction

The colorectal cancer (CRC) is the third most prevalent cancer in the world and is the fourth leading cause of death. More than a million of new cases of CRC per year are diagnosed worldwide and more than one-third of them result in death of cancer patients [1]. Alarmingly increasing number of reported cases of colon cancer in recent years has made this form of cancer a major health concern [2]. The major cases of colorectal cancers are considered sporadic, not caused by an inherited mutation; only 5–15% are caused by hereditary factors. It has been reported in different epidemiological studies that besides age and inflammation, the individual lifestyle like dietary habits, smoking, alcohol consumption and physical activity are also significant risk factors for the development of CRC [3].

Diet with high contents of red and processed meat, rich in saturated fatty acids and poor in calcium, folate, and fiber may increase the risk for colon cancer. Several studies also suggest that people consuming diets containing fewer portions of fruits and vegetables are also at a high risk for colon cancer [4]. Obesity and inflammatory bowel disease such as Crohn's disease and ulcerative colitis represent an important risk factor for CRC [5]. To date, the main CRC treatment remains surgical resection combined with chemotherapy. In recent years, there has been increasing interest to find natural products able to contribute to the fight against CRC and improve current treatment [6].

Natural polyphenols, secondary metabolites of plants involved in the defense against several types of stress, have many potential benefits in human health and reduce the risk for many cancers [7,8]. Positive effects of polyphenols are mainly attributed to their antioxidants properties supporting their anti-tumor effect which results from their direct action on malignant cell proliferation by inducing different programs of cell death including apoptosis [9,10], or indirectly by inhibiting matrix metalloproteinases [11] and vascular endothelial growth factor [12] which contribute in counteracting angiogenesis and metastasis development. Hence, natural polyphenolic compounds may be useful for prevention of cancers or may be used as chemotherapeutic agents [13].

*Olea europea* (Olive tree) belonging to the Oleaceae family is a small evergreen tree and a natural polyphenol source. The wild olive trees or Oleaster (var. *sylvestris*) and the cultivated olive trees (var. *europaea*) constitute the two botanical varieties of *Olea europaea* [14]. The Oleaster (Zebouj) is omnipresent in Algeria and widely used for different purposes. The medicinal properties of the oleaster tree are mostly attributed to the leaves used in infusion or decoction [15]. *Olea europaea* leaves have been widely used in traditional remedies in European and Mediterranean countries for their bioactive compounds. Several active phenolic compounds in unprocessed olive leaves such as oleuropein and hydroxytyrosol and other flavonoids have been identified [16].

Previous investigations carried out on oleaster leaf extracts have demonstrated their antioxidant, antibacterial, hypoglycemic, and hypocholesterolemic properties [15,17–19], but to our knowledge, no studies have been conducted on their anticancer and anti-proliferative activities.

In the present study, we investigated the effect of polyphenol-rich infusion from oleaster leaves on xenograft HCT116 tumor growth in nude mice and determined the molecular mechanism of the pro-apoptotic effect of phenolic extract from oleaster leaves (PEOL) in HCT116 and HCT8 colon cancer cell lines.

## Materials and methods

### Materials

Fresh green oleaster leaves were collected from the region of Tlemcen, Algeria. Athymic nude mice were purchased from Charles River Laboratories (France). Human colorectal cancer cell lines (HCT116, HCT8) and normal colon epithelial CCD 841 CoN cell line were purchased from American Type Culture Collection (Rockville, MD). The HCT-116 p53^+/+^ and p53^-/-^ cell lines were kindly provided by B. Vogelstein [20]. Dulbecco’s Modified Eagle’s Medium (DMEM) was purchased from Lonza Verviers SPRL (Belgium). Fura-2 AM was purchased from Life Technologies (France). Cell Proliferation Kit I (MTT) was from Sigma-Aldrich. Anti-cytochrome *c* antibody (37BA11) was purchased from abcam (France). Anti-eIF2α (5324), anti-P-eIF2α (3579), anti-P53 (2524), anti-caspase-9 (9508), and anti-cleaved PARP (9541) antibodies were obtained from Cell Signaling (France). Annexin V-APC was purchased from Biolegend (France) and 7-amino-actinomycin D (7-AAD) from BD Biosciences (Belgium). FAM-DEVD-FMK was obtained from Immunochemistry Technologies (USA). Trypsin was purchased from Gibco (USA). Tetramethylrhodamine methyl ester perchlorate (TMRM) and MitoSOX Red were obtained from Molecular Probes (France). All other chemicals were purchased from Sigma (USA).

### Extraction and determination of phenolic compounds

Fresh green oleaster leaves were collected from the region of Tlemcen, Algeria. This study was conducted on government owned property and no permission was required as per Algerian Law. The Field study did not involve endangered or protected species and no permission was required from Algerian government. The plant was recognized by a botanist (Pr Benabadji Nouri, Université Aboubekr Belkaïd, Tlemcen) of the Herbarium Center of the Faculty of Pharmacy (Tlemcen) which contained the voucher specimen (OSI 942). Phenolic extract was obtained according to the method described elsewhere [21]. Briefly, 2 g of oleaster leaf powder were extracted two times (2 h for each extraction) with 40 ml of methanol–acetone–water (7:7:6, v/v/v) at room temperature (25 ± 2°C) with constant stirring. The mixtures were centrifuged (20 min at 4000 g) and supernatants were collected and subjected to extraction with an equal volume of hexane to eliminate lipids. The total phenolic content in the extract was determined by spectrometry using ‘‘Folin-Ciocalteu” reagent assay. Briefly, 0.5 ml polyphenol extract was reacted with 2.5 ml of Folin-Ciocalteu reagent (0.2 mol/l) for 4 min, then 2 ml saturated sodium carbonate solution (75 g/l) was added into the reaction mixture. After 2 h incubation at room temperature, the absorbance at 760 nm was determined. The content of phenolic compounds was determined with reference to standard curve determined with gallic acid. The content of phenolic compounds was expressed as mg gallic acid equivalents (GAE)/g dry matter (mg GAE/g).

### *In vivo* experiments

Infusion from oleaster leaves was prepared by mixing 1 g of powder with 100 ml of boiling water for 15 min [22].

Athymic nude mice were housed under standardized conditions (20–22°C, 45–50% relative humidity) and experiments were performed in strict accordance with the recommendations in the Guide for the Care and Use of Laboratory Animals of the National Institutes of Health. The protocol was approved by the Animal Experimental Ethics Committee of the University of Burgundy (Permit Number: 2212).

10^6^ HCT116 colon cancer cells were suspended in 0.1 ml PBS and were injected subcutaneously into the left flank of nude mice (n = 20). Mice were randomly assigned the day of injection to a control group receiving tap water in feeding-bottle, and to a treated group receiving leaf infusion. Tumor volume, based on caliper measurements, was calculated during the experiment following the formula: *shortest length*^*2*^ *x largest length x 0*.*5*.

### Cells and treatment

Human colon cancer HCT116, HCT8, HCT116 p53^+/+^ and p53^-/-^ cell lines were maintained in Dulbecco’s Modified Eagle’s Medium (DMEM) supplemented with 10% heat inactivated fetal bovine serum (FBS), penicillin (100 U/ml), streptomycin (100 μg/ml), L-glutamine (2 mM). Normal human colon epithelial cells CCD 841 CoN were cultured in Eagle’s Minimum Essential Medium and supplemented with 10% FBS. Cells were incubated at 37°C in a humidified atmosphere with 5% CO_2_ and subcultured after trypsinization. For experimental conditions, cells were treated with PEOL or PBS as vehicle for 24 h.

### Cell viability assay

Cell viability was evaluated by MTT cell proliferation Kit. Briefly, HCT116 and HCT8 cells were seeded in 96-well plates at 6×10^3^ cells/well and incubated at 37°C in a humidified atmosphere with 5% CO_2_. After 24 h, the cells were incubated in 100 μl fresh medium and treated with increasing concentrations of PEOL (0, 5, 10, 20, 30, 50, and 70 μg/ml) for 24 h. Then 10 μl of MTT [3-(4, 5-dimethylthiazol-2-yl)-2, 5-diphenyltetrazolium bromide] solution (0.5 mg/ml final concentration) was added to each well and within 2 h the reaction was stopped by addition of 100 μl solubilisation reagent (10% SDS in 0.01 M HCl). After 24 hours, the absorbance was measured at 690 nm using multiwell spectrophotometer (Bio-rad). Results were expressed as % of absorbance of treated cells relative to untreated cells.

### Assessment of mitochondrial apoptotic pathway

#### Mitochondrial transmembrane potential assay (Δ*Ψ*m)

Decrease in mitochondrial transmembrane potential (Δ*Ψ*m), a marker for mitochondrial integrity disruption, is one of the earliest events that lead to apoptosis. To measure mitochondrial transmembrane potential, we used tetramethylrhodamine methyl ester perchlorate (TMRM), a fluorescent lipophilic cationic probe readily taken up by live cells and accumulated in energized mitochondria [23]. Loss of fluorescence intensity of TMRM indicates loss of Δ*Ψ*m. After 24 h treatment with PEOL, cells were incubated in medium containing 20 nM TMRM (Molecular Probes, France) for 20min at 37°C. The cells were subsequently washed three times with cold PBS, and finally recovered in 200 μl PBS. Δ*ψ*m was assessed by flow cytometry (FACSCalibur, Becton Dickinson) and data were analyzed using Flowjo software version 10 (Tree Star) results were expressed as % of cells.

#### Measurement of mitochondrial ROS

Cells were treated with PEOL (10, 20, and 30 μg/ml) for 24 hours. In some experiments, cells were incubated for 1 h with BAPTA-AM (5μM) before treatment with PEOL. After the treatment, cells were washed with PBS and then incubated with 2.5 μM MitoSOX Red (Molecular Probes, France) for 20 min, at 37°C in a humidified chamber containing 95%air and 5% CO_2_, to detect mitochondrial superoxide, primary ROS produced by mitochondria. The MitoSOX red was removed, and cells were subsequently washed twice with PBS, harvested and resuspended in 200 μl PBS. Finally, cells were analyzed by flow cytometry and data were processed using FlowJo software version 10 (Tree Star).

### Measurement of free intracellular calcium [Ca^2+^]i concentrations

HCT8 and HCT116 cells were cultured on WillCo-dish wells with a glass bottom and loaded with Fura-2/AM (1 μM) for 60 minutes at 37°C in loading buffer that contained: 110 mM NaCl; 5.4 mM KCl; 25 mM NaHCO_3_; 0.8 mM MgCl_2_; 0.4 mM KH_2_PO_4_; 20 mM HEPES-Na; 0.33 mM Na_2_HPO_4_; 1.2 mM CaCl_2_, pH 7.4 as described by Dramane et al. [24]. The changes in intracellular Ca^2+^ (F340/F380) were monitored under a Nikon microscope (TiU) by using an S Fluor 40× oil immersion objective. The planes were taken at z intervals of 0.3 μm, and NIS-Elements software was used to deconvolve the images. The microscope was equipped with an EM-CCD (Lucas) camera for real-time recording of 16-bit digital images. The dual excitation fluorescence imaging system was used for studies of individual cells. The changes in intracellular Ca^2+^ were expressed as Δ ratio, which was calculated as the difference relative to the peak F340/F380 ratio. The data were summarized from the large number of individual cells (20–40 cells in a single run, with 3–9 identical experiments done in at least 3 cell preparations). For experiments conducted in the absence of external calcium (0% Ca^2+^), CaCl_2_ was replaced by 1 mM EGTA in the buffer. All test molecules were added in small volumes with no interruption in recordings.

### Detection of apoptosis by flow cytometry

A simultaneous allophycocyanin-conjugated Annexin V (Annexin V-APC), 7-amino-actinomycin D (7-AAD) staining was used to quantitatively determine the percentage of cells undergoing apoptosis. Briefly, HCT116 and HCT8 cells were incubated or not with 50 μM N-benzyloxycarbonyl-Val-Ala-Asp-fluoromethylketone (z-VAD-fmk) for 1 h, and then treated with 20 μg/ml PEOL for 24 h. To investigate the role of [Ca^2+^]i, cells were preincubated for 1 h rather with BAPTA-AM (5 μM), or with ruthenium red (5 μM) before PEOL treatment. To investigate the role of ROS, cell were preincubated with N-acetyl-L-cysteine (NAC) 0.5 mM for 1 h before PEOL treatment. Cells were harvested and stained with Annexin V-APC and 7-AAD, and then analyzed by fluorescence-activated cell sorting using FACScan (BD Biosciences, San Jose, CA, USA). Data were processed using FlowJo software version 10 (Tree Star).

### Western blot analysis

HCT116 and HCT8 cells were cultured and treated with various concentrations of PEOL for 24 h. For whole cell extract, the cells were lysed with RIPA buffer (Thermo Fisher Scientific Inc., Rockford, IL, USA) supplemented with a protease inhibitor cocktail (Sigma). For cytoplasm extract, cell pellets were resuspended in 5 volumes of ice-cold buffer (10 mM HEPES-KOH, pH 7.9, 1.5 mM MgCl_2_, 10 mM KCl, 0.5 mM DTT, 0.5 mM PMSF, and 2 μl/ml protease inhibitor cocktail), left on ice for 10 min, and were lysed with 20 strokes of a tight-fitting Dounce homogenizers. After centrifugation (250 g, 10 min at 4°C) nuclear and cell debris was removed. The supernatant was further centrifuged at 100, 000 g for 90 min and the resulting supernatant was used for cytochrome *c* analysis. The protein content was determined by bicinchoninic acid (BCA) assay (Thermo Fisher Scientific, France). 80 μg protein of whole cell or 20 μg of cytoplasm extract samples were separated by electrophoresis on 10% sodium dodecyl sulfate (SDS)-polyacrylamide gels and transferred to polyvinylidine difluoride (PVDF) membranes. The membrane was washed twice for 10 min with Tris Buffered Saline containing 0.1% Tween-20 (TBS-T), followed by blocking with TBS containing 5% non-fat milk for 1 h at room temperature. The incubation with 1:1000 working dilution of the primary antibodies in the blocking solution was performed overnight at 4°C. The membrane was washed three times for 5 min with TBS-T and subsequently incubated with a 1:4000 dilution of the HRP- secondary antibodies for 2 h at room temperature. Anti-*β*-actin was used as loading control. After washing 3 × 5 min with TBS-T, the membrane was exposed in the enzyme-catalyzed chemiluminescent (ECL) kit (Amersham, France) on Bio-Rad ChemiDoc XRS+ system. Densitometric analysis was performed on Bio-Rad Image Lab Software (version 4.1).

### Detection of caspase-3 and caspase-7 activities

HCT116 and HCT8 cells were treated for 24 h with PEOL (10 μg/ml and 20 μg/ml), then harvested and stained by incubating for 1 h at 37°C with caspase 3/ 7-FAM-DEVD-FMK solution (Immunochemistry Technologies, Bloomington, MN, USA). After 1 h cells were washed twice with wash buffer, pellets were resuspended in wash buffer and then analyzed by flow cytometry. Data were processed using FlowJo software version 10 (Tree Star).

### RT-PCR quantification assay

Total RNA was prepared using trizol reagent (Invitrogen Life Technologies). 1 μg of total RNA was reverse transcribed with superscript II RNAse H-reverse transcriptase using oligo (dT) according to the manufacturer’s instructions (Invitrogen Life Technologies, France).

Real-time PCR was performed using the icycler iQ™ Real Time Detection system (Applied). Amplification reactions were done using SYBR Green I detection 7500 Fast Real-Time PCR System (Applied Biosystems) with the following primers as previously described [25]: CHOP: sense 5’-ACA CAG ATG AAA ATG GGG GTA CCT-3’ and antisense 5’-AGA AGC AGG ATC AAG AGT GGT CCT-3’: β-actin: 5′-CTG GTG CCT GGG GCG-3′ and 5′-AGC CTC GCC TTT GCC GA-3′. CHOP expression was normalized to β-actin and calculated using the 2^-ΔΔCt^ method.

### Statistics

Results were expressed as mean ± SEM for a given number of experiments (n). Data were analyzed by using Statistica (4.1 version, Statsoft, Paris, France). The significance of differences between mean values was determined by one-way ANOVA, followed by Fisher’s least-significant-difference (LSD) test. Differences with *p* < 0.05 were considered to be significant.

## Results

### PEOL inhibited tumor growth in HCT116-xenografted nude mice

**Fig 1:**
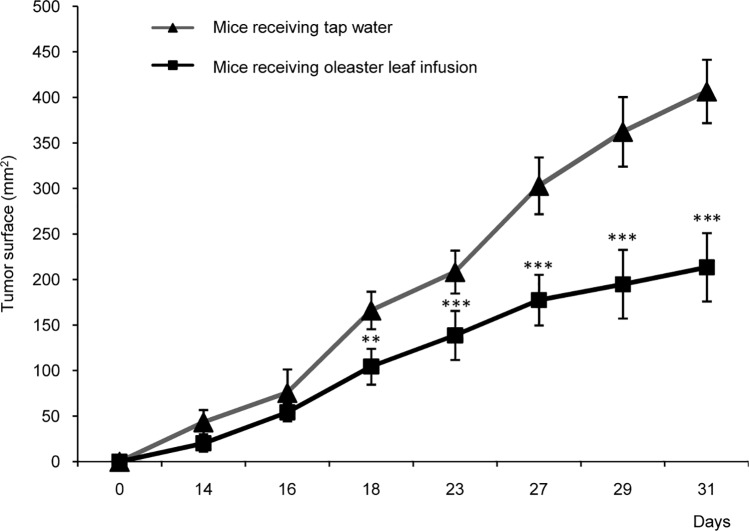
Oleaster leaf infusion decreases tumor growth in athymic nude mice xenografted with HCT116 cells. Cells (1×10^6^) were injected subcutaneously into the left flank of nude mice (n = 10). Half of these animals received oleaster leaf infusion (1%) in the feeders, while tap water was given to the other half (control). Palpable tumors were measured using a Vernier caliper for 30 days. ** and *** represent *p* < 0.01 and *p* < 0.001, respectively, as compared to group of mice receiving tap water. *p* values were obtained by one-way ANOVA, followed by Fisher’s LSD test.

The analysis of polyphenolic content of oleaster leaf was performed and oleaster leaf extraction with methanol/acetone/water revealed a total phenolic content estimated to be 71.49 mg GAE/g DW. Moreover, oleaster leaf infusion contained 0.5522 mg GAE/ml. The anti-tumor activity of PEOL was examined in HCT116 cells-transplanted nude mice. Early after HCT116 transplantation, mice received leaf infusion in feeding-bottle and HCT116 tumor progression has been evaluated. In the control group, tumors grew rapidly and reached to an average surface of 400 mm^2^ on 31 days post-HCT116 transplantation (S1 File). We found that oleaster leaf infusion diminished significantly tumor growth by two fold that reached only 200 mm^2^ after 31 days of HCT116 injection (Fig 1). The present data highlights the capacity of PEOL to limit tumor growth in nude mice.

### PEOL decreased colon cancer cell viability

**Fig 2:**
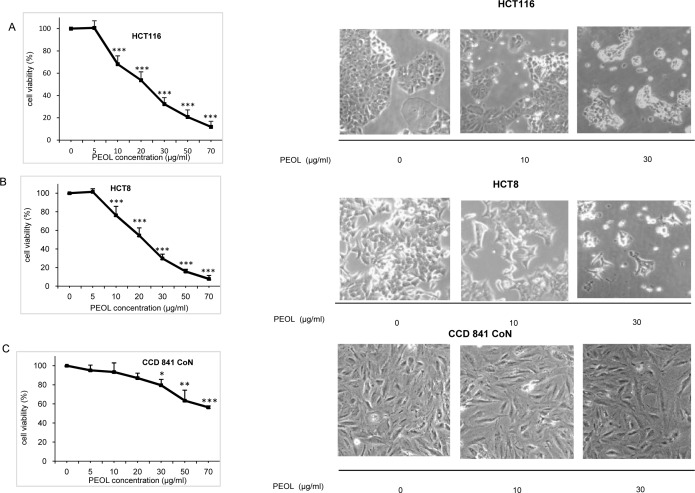
**Effects of PEOL on the viability and morphology of HCT116 (A), HCT8 (B), and CCD 841 CoN cells (C)**. The cells (6 ×10^3^ cells/well) were incubated with different concentrations of PEOL for 24 h as described in Materials and Methods. Cell viability was measured by MTT test and morphology was assessed by light microscopy (magnification ×200). *,**,*** represent *p* < 0.05, *p* < 0.01, *p* < 0.001 respectively as compared to untreated cells. *p* values were obtained by one-way ANOVA, followed by Fisher’s LSD test.

The anti-tumor action of PEOL might concern HCT116 cancer cells and/or host cells. Therefore, we analyzed the role of PEOL on cancer cells (S2 File). PEOL exhibited cytotoxic effects on HCT116 (Fig 2A) and HCT8 (Fig 2B) cells in a dose dependent manner. A PEOL treatment at 20 μg/ml for 24 h reduced number of colon cancer cells about 50% without significantly affecting viability of normal colon CCD 841 cells (Fig 2C). Hence, PEOL concentration at 20 μg/ml was preferentially used for subsequent experiments.

### PEOL induced apoptosis in colon cancer cells

**Fig 3:**
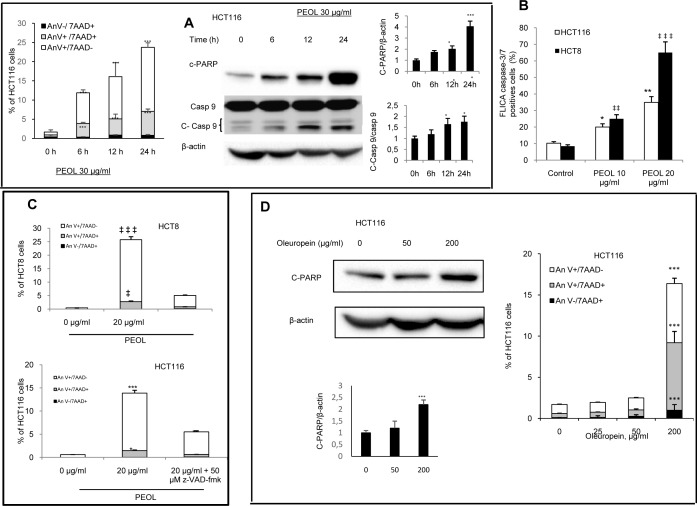
PEOL induced apoptosis in colon cancer cells. Annexin V and 7-AAD labeling, caspase-9 activation, and PARP cleavage were monitored after 6 h, 12 h, and 24 h of HCT116 exposure to 30 μg/ml PEOL (**A)**. HCT116 and HCT8 cells were treated with different concentrations of PEOL for 24 h and Caspase-3 and -7 activities were assessed by flow cytometry (**B**). HCT116 and HCT8 cells were incubated (or not) with z-VAD-fmk for 1 h, and then treated with PEOL (20 μg/ml). After 24 h, Annexin V and 7-AAD labeling was assessed by flow cytometry (C). HCT116 cells were treated with increasing concentrations of oleuropein for 24 h. then PARP cleavage and Annexin V and 7-AAD labeling were monitored were as described in Materials and methods (**D).** *,**,*** represent *p* < 0.05, *p* < 0.01, *p* < 0.001 respectively as compared to HCT116 untreated cells. ‡, ‡‡, ‡‡‡ represent *p* < 0.05, *p* < 0.01, *p* < 0.001 respectively as compared to HCT8 untreated cells. *p* values were obtained by one-way ANOVA, followed by Fisher’s LSD test.

In order to define PEOL-induced cell mortality, we analyzed apoptotic hallmarks in colon cancer cells (S3 File). We found that PEOL at 20 μg/ml induced apoptosis in a time-dependent manner evidenced by Annexin V- 7-AAD staining and expression of c-PARP and c-caspase-9 in HCT116 cells (Fig 3A). Furthermore, PEOL-treated colon cancer cells increased active caspase-3/7 content (Fig 3B) strongly suggesting the induction of caspase-dependent apoptosis. We confirmed the activation of apoptotic program in colon cancer HCT116 and HCT8 cells exposed to PEOL for 24 h since the pan-caspase inhibitor z-VAD-FMK reduced their Annexin V- 7-AAD staining compared to control cells (Fig 3C).

Oleuropein might be the major active component in PEOL. Thus, we evaluated its effect on apoptosis on HCT116 colon cancer cells. We observed that oleuropein at its maximal concentration (25 μg/ml) that can be present in PEOL (30 μg/ml) did not induce apoptosis in HCT116 cells illustrated by Annexin V- 7-AAD staining and PARP cleavage. However, we observed pro-apoptotic activity of oleuropein at higher concentration (200 μg/ml) (Fig 3D).

### PEOL-mediated apoptosis was independent of p53 status

**Fig 4:**
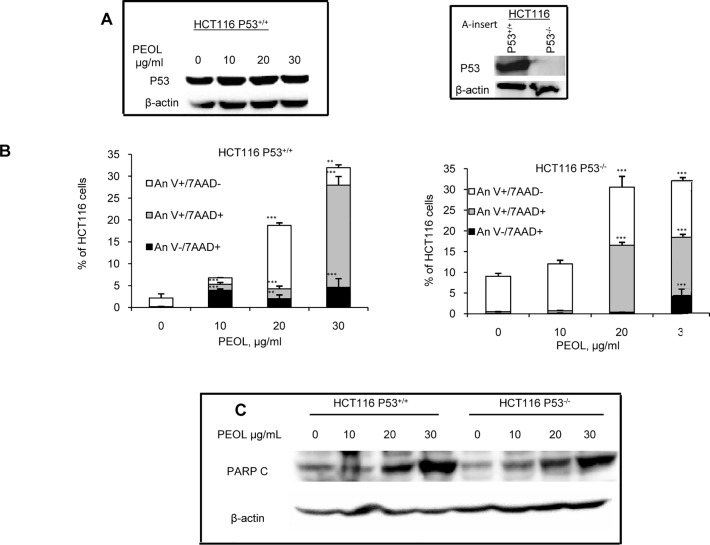
PEOL-mediated apoptosis was independent of p53 status. HCT116 p53^+/+^ and HCT116 p53^-/-^ cells were treated with increasing concentrations of PEOL for 24 h. The effect of PEOL on p53 expression in HCT116 p53^+/+^ was assessed by western blotting (A). Insert shows p53 expression in HCT116 p53^+/+^ and HCT116 p53^-/-^ (A insert). Cell death was assessed with Annexin V and 7-AAD staining (B). Cleaved PARP were determined by western blotting in cells treated with PEOL as described in Materials and methods (C). Data represent means ± SEM (n = 3). ** and *** represent *p* < 0.01 and *p* < 0.001 respectively as compared to untreated cells. *p* values were obtained by one-way ANOVA, followed by Fisher’s LSD test.

To investigate the role of p53 in cell death induced by PEOL, we analyzed the regulation of p53 expression in HCT116. We did not find any changes in p53 expression levels in HCT116 treated with different concentrations of PEOL for 24 h (Fig 4A). Furthermore, HCT116 p53^-/-^ (insert) cells and their p53^+/+^ parental counterpart exposed for 24 h to the treatment presented similar sensitivity to PEOL-induced apoptosis evidenced by Annexin V- 7-AAD staining and c-PARP expression (Fig 4B). Altogether these data indicated that PEOL triggered apoptosis in colon cancer cell lines in a p53 independent manner (S4 File).

### PEOL treatment caused loss of mitochondrial transmembrane potential (Δ*Ψ*m) and increased ROS generation

**Fig 5:**
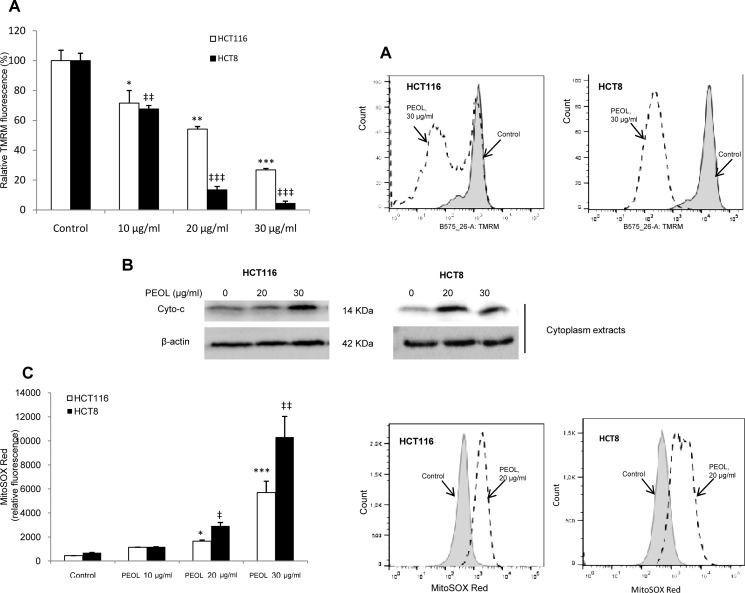
Increases in mitochondrial ROS associated with mitochondrial membrane potential (Δ*Ψ*m) decreases in PEOL-treated cells. HCT116 and HCT8 cells were treated with PEOL for 24 h, and then incubated whether with MitoSOX Red or TMRM as described in Materials and Methods. Representative histograms are shown in parallel with bar graphs showing the relative TMRM mean relative fluorescence intensity (**A**) and MitoSOX fluorescence (**C**) as measured by flow cytometry. Cytoplasmic cytochrome c was determined by western blotting as described in Materials and methods (B). Data represent means ± SEM (n = 3). *,**,*** represent *p* < 0.05, *p* < 0.01, *p* < 0.001 respectively as compared to HCT116 untreated cells. ‡, ‡‡, ‡‡‡ represent *p* < 0.05, *p* < 0.01, *p* < 0.001 respectively as compared to HCT8 untreated cells. *p* values were obtained by one-way ANOVA, followed by Fisher’s LSD test.

Irreversible mitochondrial depolarization is considered as the point-of-no-return of apoptosis and essential to activation of caspase-9. Hence, measurement of Δ*Ψ*m, by using TMRM probe showed that PEOL treatment significantly decreased Δ*Ψ*m in a dose dependent manner in HCT116 and HCT8 colon cancer cell lines (Fig 5A). Since mitochondrial depolarization induces cytochrome c release into the cytoplasm, we evaluated cytochrome c content in cytoplasm. We found that PEOL treatment induced release of cytochrome c from mitochondria into cytoplasm thereby inducing apoptosis via mitochondrial pathway (Fig 5B). Mitochondrial apoptotic pathway is mostly triggered by a decrease in Δ*Ψ*m and enhanced ROS production [26]. Thus, we observed a dose dependent increase of mitochondrial superoxide in both colon cancer cell lines treated with PEOL and incubated with MitoSOX red for mitochondrial ROS analysis (Fig 5C). These findings in agreement with the observation of caspase-9 activation suggested that PEOL triggered mitochondrial-dependent apoptotic pathway in colon cancer cell lines by decreasing Δ*Ψ*m and increasing mitochondrial ROS (S5 File).

### PEOL induced ROS-dependent ER stress

**Fig 6:**
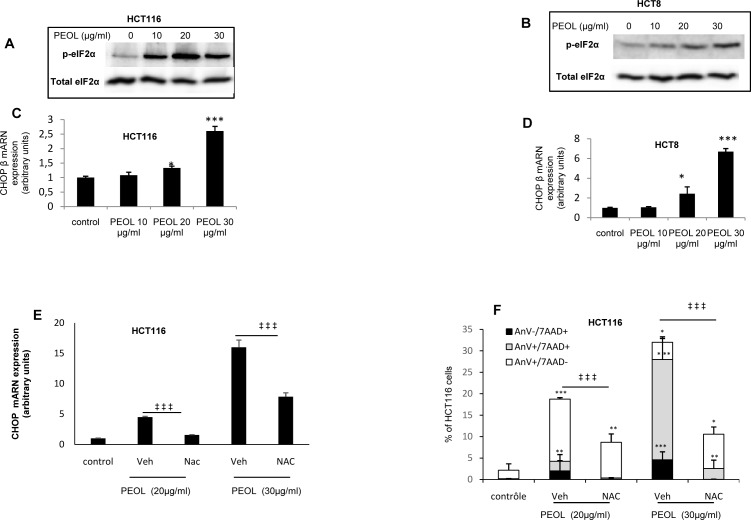
PEOL induced ROS-dependent ER stress. HCT116 and HCT8 cells were treated with PEOL for 24 h then eIF2α phosphorylation and CHOP mRNA expression were determined by western blotting (A,B) and Real-time PCR (C,D), respectively. HCT116 were treated incubated with NAC (0.5 mM) before PEOL treatment for 24 h, then CHOP mRNA expression (E) or Annexin V and 7-AAD labeling were performed (F). Data represent means ± SEM (n = 6). *,**,*** represent *p* < 0.05, *p* < 0.01, *p* < 0.001 respectively as compared to untreated cells.‡‡‡ represent *p* < 0.001 for Annexin V+ comparison between NAC-treated and untreated cells. *p* values were obtained by one-way ANOVA, followed by Fisher’s LSD test.

Endoplasmic reticulum stress (ER stress) originates from several cellular alterations such as inhibition of protein synthesis, modification of calcium homeostasis and ROS generation [25]. Increase in eIF2α phosphorylation, a translation initiator factor, and expression of CHOP, a transcription factor involved in ER stress-mediated apoptosis, is hallmark of ER stress. We found that PEOL treatment increased eIF2α phosphorylation (p-eIF2α) (Fig 6A and 6B) and CHOP mRNA expression (Fig 6C and 6D) in HCT116 an HCT8 cells in a dose dependent manner. Furthermore, the ROS scavenging with NAC in PEOL-treated HCT116 cells reduced CHOP mRNA expression (Fig 6E) and counteracted the induction of apoptosis analyzed with Annexin V/7-AAD staining (Fig 6F). These results evidenced that PEOL triggered ER stress-induced apoptosis through ROS generation (S6 File).

### PEOL increased intracellular Ca^2+^ level in HCT8 and HCT116 cells triggering mitochondrial ROS induction and CHOP expression

**Fig 7:**
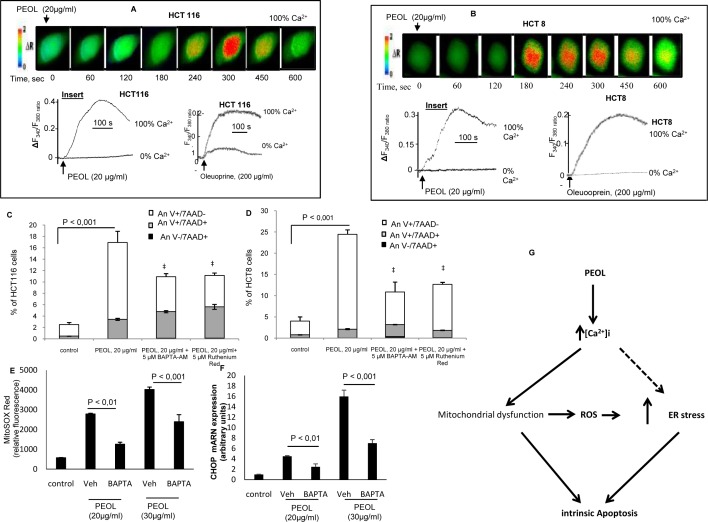
Ca^2+^ signaling modulation by PEOL in HCT8 and HCT116 cells. Cells were loaded with the fluorescent probe, Fura-2/AM. The changes in F340/F380 were monitored under a Nikon microscope (TiU) by using S Fluor 40× oil immersion objectives as described in Materials and Methods. The colored time-lapse changes in the increases in [Ca^2+^]i were recorded in PEOL-treated HCT116 and HCT8 cells in 100% Ca^2+^-buffer (**A** and **B**). The arrow head indicates the time when PEOL was added into the wells without interruptions in recordings. Inserts show the single traces of observations of cell treatment with PEOL and oleuropein in 100% or 0% Ca^2+^-buffer which were reproduced independently. (C) and (D) show the effect of cells preincubation for 1 h with BAPTA-AM (5 μM), or ruthenium red (5 μM) on PEOL induced apoptosis (Annexin V and 7-AAD staining) in HCT116 an HCT8 cell lines. (E) shows the effect of cells preincubation with BAPTA-AM (5 μM) of MitoSOX fluorescence measured after 24 h incubation with PEOL in HCT116. (F) shows the effect of cells preincubation with BAPTA-AM (5 μM) on CHOP mRNA expression in response to increasing PEOL concentration. (G) represents a schematic conclusion summarizing the molecular mechanism of PEOL-induced apoptosis. Data represent means ± SEM (n = 6). ‡ and * represents *p* < 0.01 as compared to PEOL treated cells. *p* values were obtained by one-way ANOVA, followed by Fisher’s LSD test.

The literature reported that increase in intracellular Ca^2+^ concentration ([Ca^2+^]i) induced apoptosis through the activation of mitochondrial stress and ROS production [27]. In order to define the regulation of Ca^2+^ homeostasis and the role of Ca^2+^ in PEOL-mediated apoptosis, we conducted experiments in absence (0% Ca^2+^) and presence (100% Ca^2+^) of Ca^2+^ in the extracellular medium. Fig 7A and 7B showed that PEOL induced significant increases in [Ca^2+^]i in 100% Ca^2+^ medium in HCT116 and HCT8 cells; however, PEOL exposure did not induce increases in [Ca^2+^]i in 0% Ca^2+^ medium (insert A and insert B). Oleuropein only at 200 μg/ml, also, induced significant increases in [Ca^2+^]i only in 100% Ca^2+^ medium in these cell lines (insert A and insert B). These data suggested that PEOL and oleuropein induced a Ca^2+^ influx from extracellular medium rather than its release from intracellular store. Thereafter, we analyzed the effect of BAPTA-AM, a cytoplasmic calcium chelator, on PEOL-induced mitochondrial ROS generation using MitoSOX red. We observed that BAPTA-AM addition limited the production of mitochondrial ROS in HCT116 cells treated with PEOL at 20 and 30 μg/ml for 24 h (Fig 7E). We showed that ER stress originated from ROS generation (Fig 6). Therefore, we investigated the effect of intracellular Ca^2+^ change on ER stress with CHOP expression analysis. As expected, chelation of intracellular Ca^2+^ with BAPTA-AM inhibited PEOL-mediated CHOP mRNA increase in HCT116 cells (Fig 7F). Finally, we analyzed the effect of BAPTA-AM and ruthenium red, an inhibitor of mitochondrial Ca^2+^ uptake, on PEOL-induced apoptosis. We observed that chelation and mitochondrial uptake inhibition of calcium significantly reduced PEOL-induced apoptosis evidenced by decrease of Annexin V staining (Fig 7C and 7D).

Altogether, our data demonstrated that the alteration of calcium homeostasis induced ROS production driving ER stress-activated apoptosis (S7 File).

## Discussion

Cancer cells escape programmed cell death by different strategies and reactivation of pro-apoptotic program appears as a big challenge in the anti-cancer fight. In this context, polyphenols derived from natural products might be very helpful and we showed in the present study that phenolic extract of the oleaster leaves (PEOL) has anti-tumor effect using a HCT116-xenografted mice model and pro-apoptotic properties in human colon cancer cell lines.

The olive tree, botanically known as *Olea europaea*, is rich in polyphenols and is known for its antioxidant capacity [28]. Moreover, antiproliferative effects of olive leaf extract have been noted in promyelocytic leukemic cells (HL-60) [29] and Jurkat leukemic cells [30]. However to our knowledge, the effects of oleaster leaf extract on proliferation and tumor progression have not been reported yet.

In this study, we clearly demonstrated that PEOL decreased colon cancer cell viability by inducing intrinsic apoptosis dependent on increase of intracellular calcium, mitochondrial ROS production and ER stress (Fig 7G). It is noteworthy that normal colon epithelial cells (CCD 841 Co) are significantly less affected by PEOL-decreased cell viability. Interestingly, Han et al. have previously shown that phenolic compounds of olive leaf, hydroxytyrosol and oleuropein, were effective in reducing MCF-7 human breast cancer cell viability [31]. Oleuropein is the most abundant phenolic compound in oleaster leaves which contain a higher level of polyphenols than olive oil [28]. However, purified oleuropein at 50 μg/ml was not able to induce apoptosis in HCT116 and HCT8 whereas we observed a pro-apoptotic effect of PEOL with a concentration as less as 20 μg/ml in both colon cancer cells. PEOL action on cell death relied on synergism between their different components identified for pro-apoptotic properties such as oleuropein and hydroxytyrosol but also luteolin, quercetin, chryseriol and apigenin [32]. Therefore, the pro-apoptotic program of PEOL probably is its own and may differ from mechanism of individual component found in PEOL. For instance, apigenin has been reported to regulate expression of the pro-apoptotic p53 in HCT116 cells [33] while we found that PEOL action did not modify p53 expression and PEOL-induced apoptosis was independent of p53 status. Similarly, oleuropein at pro-apoptotic concentration (200 μg/ml) did not increase p53 expression and act through p53 pathway (data not shown). However, several *in vitro* as well as few *in vivo* studies have reported that polyphenols such as resveratrol, curcumin and EGCG exhibit anticancer properties by inducing overexpression of p53 protein in several cancer cell lines [34,35].

Some anticancer agents inducing apoptosis in cancer cells are associated with a rapid collapse of mitochondrial membrane potential [36]. Our results clearly showed that PEOL treatment decreased Δ*Ψ*m as measured by flow cytometry using TMRM fluorescent dye. Δ*Ψ*m dissipation is an early stage of apoptosis that precedes DNA fragmentation, ROS production and increase of membrane permeability [37,38] and promotes activation of caspases [39]. We have previously shown that increases in [Ca^2+^]i mediated the antiproliferative effect of polyphenols [21,40]. Ca^2+^ is an intracellular secondary messenger that controls several cellular functions including apoptosis. We observed that PEOL and oleuropein (only at pro-apoptotic concentration 200 μg/ml) induced a dramatic increase in [Ca^2+^]i, in both HCT116 and HCT8 cells only in the presence of Ca^2+^ in the extracellular medium suggesting that PEOL triggered a Ca^2+^ influx probably through the opening of calcium channels.

This increase in [Ca^2+^]i could represent a mechanism by which PEOL induces mitochondrial stress and subsequent apoptosis. Indeed, mitochondrial Ca^2+^ accumulation is triggered by several stimuli that increase [Ca^2+^]i. Additionally, mitochondrial Ca^2+^ uptake during the apoptotic process leads to the opening of permeability transition pore complex (PTPC) leading to the release of apoptotic factors from mitochondria [27]. In accordance with Kroemer et al. who claimed that the progressive loss of Δ*Ψ*m is often accompanied by an increased generation of ROS, we observed that PEOL treatments dramatically increased mitochondrial ROS as assessed by MitoSOX Red dye [39]. Taking together the above findings, we investigated the role of [Ca^2+^]i increase in PEOL-induced mitochondrial ROS generation. We observed that pretreatment with BAPTA-AM significantly inhibited PEOL-induced ROS production. Furthermore, apoptotic effect of PEOL was reduced by pretreatment with BAPTA-AM and ruthenium red underlying the pivotal role of PEOL-induced [Ca^2+^]i increase in the alteration of mitochondrial function associated with apoptosis. The role of increase of [Ca^2+^]i in cancer cells apoptosis has been already described. Indeed, Kruman and Mattson reported that staurosporine led to an increase of [Ca^2+^]i and consequently, to apoptosis in neuronal cells, and chelation of intracellular calcium reversed this apoptosis [41]. Similarly, in EGCG-induced apoptosis in retinoic acid-resistant acute promyelocytic leukemia cells, there was an increase of ROS whose major sources are components of the mitochondrial respiratory chain [42].

The key role of mitochondria in the generation of primordial ATP for the survival and proliferation of eukaryotic cells has been proven by extensive biochemical studies [43]. Mitochondria also play a central role in the regulation of programmed cell death [39]. Indeed mitochondria act as central regulators of the intrinsic apoptotic pathways and effector-mediators of the extrinsic apoptotic pathways [44]. Mitochondrial depolarization induces cytochrome *c* release into the cytoplasm where it activates caspase-3 via caspase-9 by interacting with Apaf-1 [45]. The activated caspase-3 cleaves the PARP, which is one of the hallmarks of apoptosis induced by several natural products[46]. We observed that PEOL induced the cytochrome *c* exit from mitochondria followed by the cleavage of procaspase-9 into its active form leading to the activation of downstream caspases-3/7 activity and the cleavage of PARP highlighting induction of apoptosis program.

Besides the involvement of ROS in the promotion of cytochrome c release into cytoplasm via dissociation from cardiolipin in the inner mitochondrial membrane [47], ROS represent a critical signal for induction of ER stress and apoptosis. ER stress triggers an adaptive program called unfolded protein response (UPR) representing a signal induction for cell death in the case of prolonged ER stress. As far as ER is concerned, transmembrane ER protein kinase (PERK) activation represents one of the signals that initiate UPR, PERK phosphorylates eIF2α and consequently attenuates global protein translation but increases translation of Activating Transcription Factor 4 (ATF4). ATF4 in turn induces the expression of CHOP a transcription factor which increases expression of genes contributing to apoptosis program. We found that PEOL dose dependently increased the eIF2α phosphorylation and CHOP mRNA expression, both hallmarks of ER stress, leading to the activation of both the extrinsic and intrinsic pathways of cell death. As PEOL failed to activate caspase 8 (data not shown), it is unlikely that PEOL activated extrinsic apoptotic pathway. ER stress induced by andrographolide, a terpene natural product, contributes to apoptosis of HCT116 cell [48]. Therefore, ER stress induced by PEOL seemed to be mediated by ROS generation as NAC reversed PEOL-induced CHOP mRNA expression and subsequent apoptosis.

Collectively, these results support the hypothesis that the mitochondrial pathway and ER stress are involved in apoptosis induced by PEOL in colon cancer cells.

Finally, we described for the first time anti-tumor activity of PEOL diluted in the drinking water of nude mice with a HCT116 transplanted tumor cells and postulated that such anticancer properties of PEOL relied in part on intrinsic pro-apoptotic induction in cancer cells.

## Conclusion

Our results have demonstrated for the first time that the phenolic extract of the oleaster leaves induced intrinsic signaling pathway-mediated apoptosis in HCT116 and HCT8 human colon cancer cells and inhibited colon carcinoma HCT116 tumor growth. These findings suggest that PEOL of *Olea europaea* var. *sylvestrisis* might be an interesting source of chemotherapeutic agents involved in the inhibition of tumor growth.

## Supporting information

S1 File Oleaster leaf infusion reduces tumor growth in HCT116-xenografted mice. (XLSX)

S2 File PEOL cytoxicity on normal (CCD 841 CoN) and cancer (HCT116 and HCT8) colon cells. (XLS)

S3 File PEOL induces caspase-dependent apoptosis in HCT116 and HCT8 colon cancer cells. (XLSX)

S4 File PEOL induces p53-independent apoptosis in HCT116 and HCT8 colon cancer cells. (RAR)

S5 File Mitochondrial stress triggered by PEOL treatment in HCT116 and HCT8 colon cancer cells. (RAR)

S6 File Reticulum stress triggered by PEOL treatment in HCT116 and HCT8 colon cancer cells. (RAR)

S7 File Early and last events involved in PEOL-induced apoptosis in colon cancer cells. (RAR)
