# Correction: Phenolic extract from oleaster (*Olea europaea* var. *Sylvestris*) leaves reduces colon cancer growth and induces caspase-dependent apoptosis in colon cancer cells via the mitochondrial apoptotic pathway

**DOI:** 10.1371/journal.pone.0176574

**Published:** 2017-04-20

**Authors:** Wafa Zeriouh, Abdelhafid Nani, Meriem Belarbi, Adélie Dumont, Charlotte de Rosny, Ikram Aboura, Fatima Zahra Ghanemi, Babar Murtaza, Danish Patoli, Charles Thomas, Lionel Apetoh, Cédric Rébé, Dominique Delmas, Naim Akhtar Khan, François Ghiringhelli, Mickael Rialland, Aziz Hichami

There is an error in the XML that is causing the fourteenth author’s name, Naim Akhtar Khan, to be indexed incorrectly. The name should be indexed as Khan NA and not Akhtar Khan N.
